# Supplementary material for: Comparative transcriptome analysis of papilla and skin in the sea cucumber, Apostichopus japonicus
Source: PeerJ. 2016 Mar 7;4:e1779. doi: 10.7717/peerj.1779 (PMC4793329; doi:10.7717/peerj.1779)
Supplement: Supplemental Information 1 [file peerj-04-1779-s001.pdf]

Table S1. PCR primers used for qRT-PCR validation.

No.	Gene ID	Gene name	Annealing temperature	Primers sequences	
				Sense5'-3'	anti-sense5'-3'
1	gi|72018933|ref|XP_797207.1|	AAC-rich mRNA clone AAC4 protein-like	55℃	TACAGAATGCCCGACAGAACG	CGCCACGATGATTAACACGAC
2	gi|325301261|gb|ADZ05534.1|	heat shock protein 26	61℃	TCCATCATTTGGCCTTCAGCGTA	CATCAAACTCGTCATCGGCAAGC
3	c75753.graph_c0	novel protein	55℃	TTGGAAACAGGAAGAGGGAGAT	TGGGGAGTGGGAATTAGCAT
4	gi|668583121|gb|AIH07018.1|	Tenascin	55℃	GAACGGTGACTACAACGCTTATT	CGTATCTTCATCTCGCTGTATTTG
5	PF07546.8	EMI domain	61℃	GTGGTGGATTCGGAGGTGA	CTGTCGCCATTTCTGCTTTC
6	gi|669214824|emb|CDW61111.1|	hypothetical protein TTRE_0000953901	55℃	CTTAGGCTTTCGGTGGACAG	GTAAGGGCGACGAAGGTAGAT
7	gi|260784620|ref|XP_002587363.1|	Fibrinogen-like protein A	55℃	GGTGGGGGAGGGTTGTTT	GGTGGTTCAATGCCTGTGG
8	gi|675363604|gb|KFM56506.1|	hypothetical protein X975_24482	55℃	ATACCCAAGTCAGACGAACGATT	GGACCCGAAAGATGGTGAACT
9	gi|195035349|ref|XP_001989140.1|	Serine/threonine-protein phosphatase	55℃	ATGAGTCACGGCAGATCACA	GCGGTCCAGGGCTCTTATAT
10	gi|585660669|ref|XP_002734685.2|	ficolin-2-like	55℃	CGTGTTGATGGAAGTGTTGA	TCCGCTGTAGGTTCCAAGTT
11	gi|47551003|ref|NP_999675.1|	Collagen alpha-2	61℃	CCCAAGACAGTACTCCAACATC	CGTCGTTGTAGTCAATCTTCGTG
12	gi|167683054|gb|ABZ91669.1|	Actin	61℃	TGATGTCACGCACGATTTCC	ACACAGTGCCCATCTACGAGG
13	gi|198423660|ref|XP_002129293.1|	Fibroblast growth factor	57℃	GTGGAGTCTTGCAGGCTGTAG	GAGGAGTTCGTCATGAAGCTG
14	gi|443729673|gb|ELU15512.1|	Fructose-bisphosphate aldolase	57℃	CGCACATTCCGTGACATCC	CCGACAGCCCATCAAGACC
15	gi|260827674|ref|XP_002608789.1|	Integrin alpha 2	57℃	CGTTTGGTTACGCCCTCTC	CTGATTGGTTTAGTATCGCTTGC
16	gi|260802726|ref|XP_002596243.1|	Mitotic spindle assembly checkpoint protein	55℃	GCTGGTGGCTGATTTCTTCTCC	TGGGGTCTGTGGTCTGCTGA
Reference	GenBank: EU668024.1	β-actin Ⅰ	Variable	CATTCAACCCTAAAGCCAACA	TGGCGTGAGGAAGAGCAT
